# Discrimination, disadvantage and disempowerment during COVID-19: a qualitative intrasectional analysis of the lived experiences of an ethnically diverse healthcare workforce in the United Kingdom

**DOI:** 10.1186/s12939-024-02198-0

**Published:** 2024-05-23

**Authors:** Mayuri Gogoi, Irtiza Qureshi, Jonathan Chaloner, Amani Al-Oraibi, Holly Reilly, Fatimah Wobi, Joy Oghogho Agbonmwandolor, Winifred Ekezie, Osama Hassan, Zainab Lal, Anuj Kapilashrami, Laura Nellums, Manish Pareek, on behalf of the UK-REACH Study Collaborative Group Members, Laura Gray, Anna L Guyatt, Catherine Johns, Chris I McManus, Katherine Woolf, Ibrahim Abubakar, Amit Gupta, Keith R Abrams, Martin D Tobin, Louise Wain, Sue Carr, Edward Dove, Kamlesh Khunti, David Ford, Robert Free

**Affiliations:** 1 Department of Respiratory Sciences, University of Leicester, Leicester, UK; 2 Development Centre for Population Health, University of Leicester, Leicester, UK; 3 Lifespan and Population Health, School of Medicine, University of Nottingham, Nottingham, UK; 4 The Nottingham Centre for Public Health and Epidemiology, University of Nottingham, Nottingham, UK; 5 Public Health Institute, Liverpool John Moores University, Liverpool, UK; 6 David Evans Medical Research Centre, Nottingham University Hospital NHS Trust, City Hospital, Nottingham, UK; 7 Diabetes Research Centre, University of Leicester, Leicester, UK; 8 Centre for Ethnic Health Research, University of Leicester, Leicester, UK; 9 School of Health and Social Care, University of Essex, Colchester, UK; 10 Centre for Global Health & Intersectional Equity Research, University of Essex, Colchester, UK; 11 College of Population Health, Health Sciences Centre, University of New Mexico, Albuquerque, NM USA; 12 Department of Infection and HIV Medicine, University Hospitals of Leicester NHS Trust, Leicester, UK; 13 NIHR Leicester BRC, Leicester, UK; 14 NIHR ARC East Midlands, Leicester, UK

**Keywords:** Intersectionality; intrasectionalism, Discrimination, Disempowerment, Disadvantage, Healthcare workers, COVID-19 pandemic

## Abstract

**Background:**

Healthcare workers (HCWs) in the United Kingdom (UK) have faced many challenges during the COVID-19 pandemic, some of these arising out of their social positions. Existing literature explicating these challenges (e.g., lack of appropriate PPE, redeployment, understaffing) have highlighted inequities in how these have been experienced by HCWs based on ethnicity, gender or, job role. In this paper, we move a step ahead and examine how the intersection of these social positions have impacted HCWs’ experiences of challenges during the pandemic.

**Methods:**

We collected qualitative data, using interviews and focus groups, from 164 HCWs from different ethnicities, gender, job roles, migration statuses, and regions in the United Kingdom (UK) between December 2020 and July 2021. Interviews and focus groups were conducted online or by telephone, and recorded with participants’ permission. Recordings were transcribed and a hybrid thematic analytical approach integrating inductive data-driven codes with deductive ones informed by an intersectional framework was adopted to analyse the transcripts.

**Results:**

Thematic analysis of transcripts identified disempowerment, disadvantage and, discrimination as the three main themes around which HCWs’ experiences of challenges were centred, based on their intersecting identities (e.g., ethnicity gender, and/or migration status). Our analysis also acknowledges that disadvantages faced by HCWs were linked to systemic and structural factors at the micro, meso and macro ecosystemic levels. This merging of analysis which is grounded in intersectionality and considers the ecosystemic levels has been termed as ‘intrasectionalism’.

**Discussion:**

Our research demonstrates how an intrasectional lens can help better understand how different forms of mutually reinforcing inequities exist at all levels within the healthcare workforce and how these impact HCWs from certain backgrounds who face greater disadvantage, discrimination and disempowerment, particularly during times of crisis like the COVID-19 pandemic.

## Introduction

Healthcare workers (HCWs) were found to be at an increased risk of infection from the SARS-CoV-2 virus when the COVID-19 pandemic started in 2019 [1]. Evidence also indicates that in many countries, ethnic minorities were at greater risk of infection from the virus [2]. In the United Kingdom (UK), it was found that ethnic minority populations had poorer health outcomes and higher mortality rates than White ethnic groups, particularly during the early phases of the pandemic [3, 4]. This disproportionate impact among ethnic minorities was also reflected in the healthcare workforce, with ethnic minority HCWs accounting for 63% of deaths in early 2020, as compared to their 22.3% representation in the workforce [5, 6]. Explanations for ethnic minority HCWs being disproportionately affected by COVID-19 relate to a combination of work-related risks including exposure in the line of their work, redeployment, and working in patient-facing roles or in COVID-19 settings [7, 8]. Additionally, lack of and limited access to appropriate personal protective equipment (PPE) added a further layer of complexity and challenge to staff working with COVID-19 patients, with evidence of inequities in adequate provision of PPE among ethnic minorities [9].

In countries like the UK and US, although the healthcare workforce is getting increasingly diverse but healthcare systems are not inclusive enough and disparities exists between staff groups [10]. In these countries, disadvantages and discrimination against healthcare staff, particularly from ethnic minority backgrounds, have historically prevailed and intensified during the pandemic. Previous studies have found how HCWs from ethnic minority backgrounds in the UK and US, are over-represented in lower paid roles and have faced discrimination in career progression, inadequate pay, stressful working conditions, exploitation by managers and also how they are more likely to encounter formal disciplinary procedures as compared to their White counterparts [10–15]. The Workforce Race and Equality Standards (WRES), published annually by the UK’s National Health Service (NHS) since 2016, have consistently reported significantly high rates of harassment, bullying and/or abuse experienced by ethnic minority HCWs from patients and staff members alike [16].

While factors such as exposure at work, lack of PPE and racism may have contributed to inequities in adverse COVID-19 outcomes for ethnic minority HCWs, there is limited understanding of how multiple intersecting social identities, apart from ethnicity, can affect COVID-19 experiences and outcomes. Previous studies have largely treated the workforce as a homogenous group or alternately, been informed by a diversity analysis, where gender, ethnicity or occupational role are examined in isolation [17, 18]. This can result in inadequate recognition of how various intersecting social categories such as gender, ethnicity, occupational role, age, migrant status and others can impact on individuals’ experiences and influence outcomes [19]. Intersectionality provides a framework where rather than giving primacy to one social category, the intersections of these categories are examined more closely in order to gain a better understanding of people’s experiences [20]. Previous research has applied this framework to examine the health workforce’s experiences of COVID-19 management at their workplace, and gone beyond ethnicity to elucidate the interactions of socio-economic status, nationality, migrant status and professional role among others as determining disparities in risk experienced by HCWs [7]. Whilst the concept of intersectionality acknowledges structural and systemic oppression, at times its operationalisation in research doesn’t explicitly examine the interaction between the social categories and the different ecosystem levels.

Considering the circumstance of an increasingly diverse National Health Service (NHS) workforce in the UK [21] this paper aims to understand how HCWs have been impacted by the pandemic in relation to their social positions by utilising an intersectional approach. Furthermore, it examines how the power dynamics and interaction within and between these social categories (such as ethnicity, occupational role, age and gender) at multiple ecosystemic levels have an influence on HCWs’ experiences [20, 22].

## Methodology

### Intersectionality, intrasectionalism and the methodological approach of this study

**Fig. 1:**
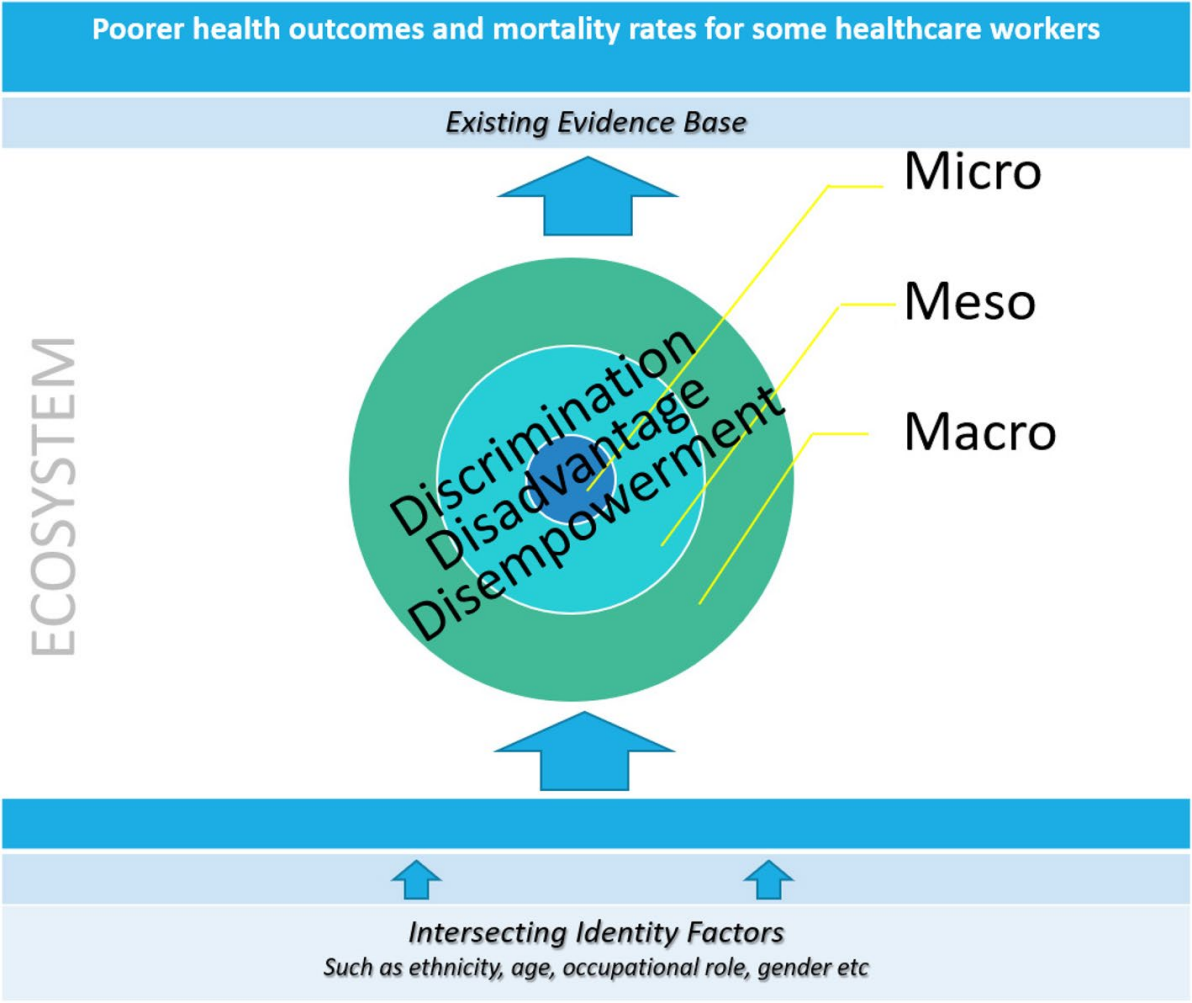
An intrasectional model for analysing HCW experiences during COVID-19

Our methodological approach to this study is grounded in the existing literature evidencing a disproportionate impact of COVID-19 on some HCWs based on social characteristics. However, our qualitative analysis is not only underpinned by an intersectional framework but also by interrogating individual experiences at an ecosystemic level [23]. It acknowledges that experiences of marginalisation or structural discrimination can be compounded not only across multiple social categories (such as ethnicity, gender and occupational roles etc.) but also across multiple systemic levels (micro, meso and macro levels) impacted by these intersecting social categories. These factors intersect with each other within a wider ecosystem, with prevailing inequities in institutions and structure which results in both horizontal (‘intersectional’ across characteristics) and vertical (‘intrasectional’ within characteristics) processes of oppression (see [24]). This overlaying of an ecosystemic approach over an intersectional framework is what we have termed ‘intrasectionalism’ in this paper (Fig. 1).

Intrasectional analysis acknowledges the combined impact of oppression at macro, meso, and micro levels and can help us more clearly identify social systems, structures and ideologies that impact individuals in their lived experience. As a set of examples, at the macro level, systemic inequities describe the ‘permeation’ of discrimination, disadvantage or disempowerment throughout society (macro/societal level). An organisation may implement this systemic inequity through a policy which disadvantages a group of individuals, this may be considered as institutional racism (meso/organisational level). An individual from that group may face aspects of everyday racism with colleagues within that organisation (micro/individual level). We propose that this intrasectional perspective on identifying differing disadvantaging factors within multiple levels is a helpful framework.

## Methods

### Setting and recruitment

This paper reports findings from the qualitative sub-study of the United Kingdom Research study into Ethnicity and COVID-19 outcomes among Healthcare workers (UK-REACH) [25]. HCWs were defined as clinical and non-clinical staff who were 16 years and over and working in a healthcare setting. HCWs from across the four devolved UK nations (England, Wales, Scotland and Northern Ireland) were invited to take part. Recruitment was through invitation emails sent out via NHS Trusts, private health contractors, professional bodies, partner organisations, Twitter advertisements, and the Professional Expert Panel (PEP) and UK-REACH stakeholder group (STAG). Participant information sheets containing study details were shared with prospective participants before start of study procedures.

### Data collection

Data collection took place online between December 2020 and July 2021. Participants underwent an online consent procedure and supplied demographic data through the same platform. Recruitment was then guided using purposive sampling to include workers from various staff grades, job roles, ages, gender, ethnicities, migration statuses and UK nations. With regards to conceptualising terms such as ethnicity, it has been previously argued that the use of ethnicity in health research has developed from an ‘untheorised’ approach where culture is mapped on to ethnic categories and ‘essentialised’ [26]. Ethnicity is a complex and contested concept, with definitions encompassing various shared characteristics including biology, culture, language and religion. This study has utilised the higher order ethnic group classifications of the Census, as used by the Office of National Statistics (ONS) in the UK (see Table 1). The higher order ethnic group classifications have also been used to minimise risk of identification of participants from the presented data [27].

**Table 1:** Participant characteristics

Variable	Total (*N* = 164)
Male	63 (38%)
Female^a^	101 (62%)
Age, median (IQR)	42 (32–53)
Ethnicity	
Asian	65 (40%)
Black	29 (17%)
Mixed	15 (9%)
White^b^	49 (30%)
Other^c^	6 (4%)
Job Role	
Doctors	44 (27%)
Nurses & Midwives	30 (18%)
Allied Health Professionals (AHP)^d^	62 (38%)
Ancillary Health Workers (AHW)^e^	28 (17%)
UK Region	
England	140 (85%)
Scotland	7 (5%)
Wales	3 (2%)
Northern Ireland	10 (6%)
Unknown	4 (2%)

^a^One participant who identified as Other was randomly assigned to the Female category to prevent identification

^b^Includes White British, White Irish, White Gypsy/Traveller and White Other

^c^Includes Arab and any other ethnic group

^d^Also includes dentists, pharmacists, healthcare scientists, ambulance workers and those in optical roles

^e^Includes those in administrative, or other non-clinical roles (e.g. housekeeping/security/maintenance etc.)

One hundred three interviews (Int) and 16 focus groups (FG) were conducted remotely via Microsoft Teams or telephone (for interviews only). A topic guide, developed in consultation with PEP and STAG was used for data collection. The topic guide (see supplementary material) was piloted and refined iteratively during data collection to ensure it was relevant and current as new key issues were identified. Interviews lasted between 45 and 60 min and focus groups took about 1.5 h. Following their participation, a gift voucher was given to HCWs in recognition of their contribution to the research. Interviews and focus groups were conducted by FW, MG, AAO, OH, IQ and LBN, who represent a range of ethnicities, and are trained qualitative researchers with experience of working with diverse ethnic and cultural groups. Discussions were recorded with permission, transcribed, and anonymised prior to analysis. Further details of methods can be found in our previous publication [25].

### Data analysis

We undertook a hybrid thematic analysis wherein data-driven inductive coding was supplemented by a theoretical coding framework informed by an intersectional framework [28]. This approach ensured that the principles of intersectionality acted as a lens (i.e. examining the intersections of HCWs social positions such as ethnicity, gender, job role and migration status) for the researchers while looking at HCWs’ experiences and allowing the themes to emerge from the data inductively [28]. The research team (MG, IQ, JC, AAO, and LBN) began with each member reading a set of transcripts to build familiarity. Thereafter, JC, MG and IQ undertook open coding of the HCW experiences simultaneously with deductive coding of intersecting HCW social positions and identified a preliminary set of codes which was mapped onto a Word document to aid collaborative analysis of the data. We adopted Nadal et al.’s [29] methodology while identifying the codes and considered an experience to be intersectional when (a) participants had explicitly stated that they felt their experience was a result of more than one identity characteristic (e.g., being a Black Muslim nurse), or (b) where based on the interview or FG, the research team had sufficient information to interpret the participant’s experience as an outcome of intersecting factors. In cases where the intersections were identified by the research team, these interpretations followed careful consideration of the context, demographic characteristics of the participants and rereading of the transcripts by more than one team member. The team discussed the coding framework and quotes within each code periodically, and JC, MG and IQ regularly updated the framework in tandem with newly identified codes as they looked at additional transcripts. Finally, when all the transcripts had been coded, the team had joint discussions to interpret the mapped data, and rearrange categories, collapse codes, and identify connections among codes to arrive at the themes and sub-themes. The team developed the final set of themes, once data saturation had been agreed, and through regular discussions and iterations (including checking back themes with participant stakeholder groups) and report these as per Consolidated criteria for Reporting Qualitative research (COREQ) guidelines.

### Reflexivity

Using intersectionality, and our model of intrasectionalism, as frameworks for analysing participant data was a deliberate strategy on the part of the research team. Therefore, it was incumbent upon us as researchers to be reflective about the potential influence of our biases and lived experiences in utilising this approach to data analysis. Intersectionality explicitly concerns itself with difference, power and inequity. We were cognisant of the diversity in the research team with members representing a variety of different ethnicities, genders and occupational experiences. We reflected on how our diversity may have influenced data collection and analysis. For example, when participants reported concerns pertaining to racialised experiences we felt there was a degree of relatability with the researchers due to perceived similarity of background. Members of the research team reflected on how shared experience may have contributed to trust or comfort, with participants being more open when discussing potentially sensitive issues.

## Results

One hundred sixty four HCWs from different roles, settings and UK regions took part in the study. A detailed breakdown of the participants’ demographic characteristics is provided in Table 1.

Thematic analysis of the transcripts identified three broad themes and several sub-themes around participants’ experiences influenced by their intersectional social positions. These themes and sub-themes have been elaborated, and interspersed with participants’ quotes, in the following paragraphs.

An intrasectional model recognises that micro, meso and macro levels aren’t mutually exclusive, as examples of discrimination, disadvantage or disempowerment can cut across, overlap and sit in between the differing levels, and can be experienced at multiple levels.

### Theme 1: Disempowerment

Under this theme we explore how ethnicity, gender, age, and migration status impinge upon experiences and perceptions of disempowerment and powerlessness among HCWs. This theme reflects an element of conscious ‘taking away’ from HCWs’ sense of authority, control, choice and influence over their own professional lives. HCWs reported feeling a loss of power or agency over the responses and decisions made in their workplaces in response to COVID-19. The disempowerment that some HCWs have faced historically and during the pandemic have nuances or layered meanings which were strongly expressed in relation to (re)deployment practices, burden on healthcare delivery, and limited agency in key decision-making about their work and well-being.

### Unfair redeployment or change in duties

Due to staff shortages and a surge in patients, staff had to be redeployed to other specialities and wards where there was more urgent need and demand. While the need to redeploy staff was justified, some staff members felt that the process of identifying staff members for redeployment was not fair. They felt that selection was not made on the basis of their skill-set, merit or preference, but was based on the social position of HCWs, most notably, ethnicity, age and, job grade/seniority. Some redeployed staff also said that they felt like there was not enough consideration for their personal circumstances or their safety and protection while redeploying them or making changes to their duties, which made them feel that they were ‘disposable’. An ethnic minority participant spoke about redeployment of ethnic minority staff from certain cultural backgrounds, and their non-disputing approach to work which puts them in a position of submissiveness:

*So it’s always the BAME* [Black, Asian and minority ethnic] *staff really that have been moved…we are sent to the Covid – like some of my colleagues are sent to the Covid wards or sent to intensive care in other hospitals. So, I just noticed the pattern really. It’s just like Filipinos, in the end it’s just mostly BAME. I think it’s just in our culture, we just keep on saying yes and if an English nurse has been sent off, they will just say blink until they say no. But ask Filipinos and other Asians, we just say ‘OK, that’s fine’. We won’t say no.* (P4, Female, Other, Nurse/Midwife, FG)

Another participant also spoke of the perceived unfair redeployment practices based on people’s ethnicity and migrant status that she had witnessed at her place of work:

*And I know that my white counterparts got a better deal out of the redeployment…my white colleagues who are fit and healthy, who were not redeployed, who were sent to areas where they were going to have a much easier life than on the Covid side. And I think that that is structural – they were management decisions and I think people’s own unconscious bias and prejudice made them absolutely 100% divert people of colour to those high-risk areas, because they’re not going to speak up, especially if you’re an immigrant and you want your job, you’re just not going to do it, it’s just not going to happen.* (P5, Female, Asian, Nurse/Midwife, Int)

### Reduced agency

A sense of agency is when people feel responsible for the decisions they make or the actions they take in their own lives. This free will and independence are hindered when the power to decide is taken away from them or diminished. HCWs in our study expressed numerous ways in which they felt that the control of deciding what was best for themselves had been taken away from them. A common manifestation of a lack of agency perceived by HCWs was ‘not being heard’ or ‘being muted’ by colleagues and employers while making decisions. In our research, multiple intersecting factors of ethnicity, gender, age, migration status, and hierarchy of roles seemed to have led to conditions where participants felt they were not able to voice their opinions. As a young female junior doctor from a mixed ethnic background remarked:

*If I was a white man, would they listen more and if I didn’t look like – I think when we go to meetings and things, I’m pretty sure they assume I’m younger than I am and so yeah, I guess I do wonder like if I was a white man saying this same thing, would you listen more? And would I also not have to say it in such a roundabout overly polite way and you would hear it.* (P1, Female, Mixed Ethnicity, Doctor, FG)

Participants often attributed their sense of disempowerment to one aspect of their identity (e.g., gender, ethnicity, migration status), but an intersectional lens reveals that there are multiple factors that could have added to this feeling of lack of agency. For example, a young ethnic minority HCW in a relatively junior role described how he felt his ethnicity, religion and role could have influenced his experiences:

- Respondent: *I’ve raised complaints, if I’ve gone to report one of my work colleagues through another member of staff, they’ve always said you should have not done that, like I’ve reported someone doing something wrong. The matrons don’t listen to you. So, it’s not just at that level, it’s beyond that. They just don’t want to listen.*
- Interviewer: *And what does that make you feel like their reason for not listening is?*
- Respondent: *The colour of my skin, I think, that’s what I think. It’s because I’m brown and probably because I pray five times a day, because I’ve got a beard. I think all of that.* (P2, Male, Asian, Nurse, Int)

Lack of opportunities to approach or question colleagues, line managers or management about practices or actions which they perceived to be discriminatory or prejudiced also made HCWs feel powerless and less in control of their own lives. Such conditions reinforce a ‘culture of silence’ among staff who largely remain silent out of fear of retribution. A young, part-time HCW said how he felt reluctant to approach senior staff at his workplace:

*I feel a bit of apprehensiveness bringing anything up to my managers, to be honest, unless it’s like a really serious issue, I don’t feel like bringing it up because then I feel like I’m bringing unnecessary attention to myself unless it’s causing me a major inconvenience or impinging my ability to do my job… Some managers aren’t very approachable and also because I’m a part time member of staff I don’t necessarily feel – and also a younger member of staff – I didn’t necessarily feel that my concerns would be taken seriously.* (P3, Male, Asian, AHP, Int)

### Burden of healthcare delivery

HCWs across the country felt under pressure to deliver healthcare during the pandemic, but the extent, form and intensity varied for different HCWs depending on their circumstances. For example, one female HCW who was part of an understaffed team of community-based mental health professionals and a single mother of a very young child, spoke of the pressures in her professional and personal life:

*So our team…I’m the only* [specialist professional] *there at the minute, but there should usually be two…but we’re understaffed…So whilst we always maintain the responsibility for service users, there are usually other people that are involved in their care anyway, so they are getting seen. So it’s* [closure of these services] *put the pressure on us and me a lot more. And then it was just the little things like I returned to work after maternity, and at the time I didn’t want to return back to work, I felt really guilty about returning back to work and leaving my little* [child]. *It was a fight within me but I literally had no choice in terms of money, so I had to return back to work.* (P6, Female, Mixed, AHP, Int)

### Theme 2: disadvantage

Another major theme is the ‘disadvantage’ that HCWs faced on account of their intersecting social positions. These disadvantages arose out of HCWs’ unique social situations, and the barriers they experienced, which limited their success or progression. While these disadvantages are embedded in social processes and had been encountered by HCWs even before the pandemic, some of these were amplified or transformed as the pandemic progressed. The disadvantages were mainly observed in the: insecurities, restrictions and precarity; limited social support; and a sense of alienation faced by HCWs at certain intersections.

### Insecurities, restrictions and precarity

Despite perceptions of heightened risks and burden, participants from minority ethnicities continued to work and stay citing insecure work, temporary contracts and income as important factors guiding their decision. HCWs who were on limited-term visas or sponsorship visas experienced restrictions in their roles and workplaces because of their migration status. Added to the insecurities and financial burden of securing visas for themselves and their families, many migrant ethnic minority HCWs had family responsibilities in both the UK and abroad, which creates conditions of precariousness that necessitate some to stay in their jobs despite being exploited or taken advantage of.

*My BAME colleagues…they were worried about their work visa expiring, so that brought a lot of stress…I have noticed that they were in that depressive state where they would be worried about ‘how will I be extended, will I still be accepted by the Trust, will I be deported,’ that sort of thing, because it all ties up to our visa, it all ties up to your contract, because we’re all international nurses.* (P7, Male, Asian, Nurse, FG)

Some HCWs also shared how their precarious financial circumstances made them cut-short their maternity or sick leave. For instance, a self-employed HCW who had to take time-off because of long COVID symptoms described her situation:

*Since I developed symptoms, COVID, and I was forced to, sort of, take more time off work, because obviously, I’m still struggling, I’m not 100% yet…I’m home, I’m off sick, and my poor colleagues are having to work above and beyond the call of duty extra hard to see not only their patients but my patients to try and meet these targets that have been imposed, and I’m home, and of course, because I’m self-employed, if I don’t work, I don’t get paid. I have to apply for NHS sick pay, which doesn’t kick in until I’ve been off sick for four weeks. So, now, technically, I can apply for it, and I don’t know how they work out how much they pay me, I don’t know if they’re going to pay me for just two weeks. I have no idea, but I have tried to push myself to go back to work prematurely, because I’m afraid of not having an income, and I just couldn’t do it.* (P8, Female, Black, AHP, Int)

Lack of choice arising out of intersecting identity characteristics was also experienced by some HCWs in other areas such as PPE accessibility. For example, one HCW recounted an incident where an ethnic minority, non-clinical, HCW was denied PPE:

*I did witness early on a black cleaner getting cross with a white matron because they did not have access to the same PPE and a not very helpful structural systems argument was used by a very well-meaning matron to say, effectively, ‘well I’m not your boss and my allocation of PPE is for the people that I’m the boss of and I’m sorry but I’m not going to be able to solve your problem.’* (P9, Male, White, Doctor, Int).

### Limited social support

Disparities in social support available to HCWs during the pandemic were reported by several participants. For example, support with childcare was a significant challenge during the pandemic and HCWs who did not have family support, for example first-generation migrants and single-parents found it difficult to balance their frontline roles with their family responsibilities.

*So, my* [child’s] *father is in* [place A] *and my family’s in* [place B]…*at the time of COVID, I’m a single mum working 100% of the time, so full time, with very thin support network other than my friends…I was barely sleeping, I was working until 2 am, I was not fun, my* [child] *was crying at nursery, not wanting to come home with me, because I had no time for* [my child], *no time for myself.* (P10, Female, White, AHW, Int)

Another HCW who was a single mother with young children shared:

*So, I mean, in terms of support, I can’t…I don’t really feel like we’ve had much support for things that were going on…There was no consideration for the fact that we’re now juggling home-schooling, no childcare…so, it’s that balance of, do you then just give in and just take the time off work, knowing financially I can’t do that, but then, how do I juggle childcare?* (P11, Female, Black, Nurse/Midwife, Int)

Speaking of the lack of a support system for some HCWs during distressing times, and misconceptions around ethnic minority support networks, one participant remarked:

*Unlike the popular myth in this country that Asian families are, you know, multigenerational, they’ve got very cohesive family units, they’ve got a lot of support in the family unit to fall back on – that is true, that is true to a large extent – but amongst healthcare workers the vast majority of background is that we are first generation in this country and, as first generation, you do not have that support. Your support is overseas, your family is overseas. And that is something that we noticed right across particularly in the first wave, we had nurses and doctors who were admitted to the ICU, who didn’t have a family that we could actually speak to, so quite a few of them were alone in this country…We have assumed that when a healthcare worker dies on the ICU, there is a family network to support them, that is definitely not the case.* (P12, Female, Asian, Doctor, Int)

### Difference and alienation

Alienation or a lack of sense of belonging was expressed by HCWs from minority background in relation to religion, ethnicity, sexual orientation, migration status etc. Several HCWs said that they had not faced any overt discrimination at their workplaces, but they could sense that they do not fit in with their colleagues, which at most times impedes their social relationships at the workplace and inhibits them from having a fulfilling work experience.

*You will realise they don’t like you because of your sexuality or because you are coming from another country - and I have stuff like that – but how I say it, I get used to* [it] *so for me it’s not something like new.* (P14, Male, White, AHW, Int)

*I mean, not directly, but you do feel some – I don’t know – I do feel that I don’t belong a lot of the times, like people would not be as close to me as they would have if I weren’t from a different background.* (P13, Female, Asian, AHP, Int)

### Theme 3: discrimination

#### Microaggressions

Participants reported the everyday unrelenting oppression of facing microaggressions, which have been characterized as subtle and sometimes unintentional acts of discriminatory behaviour which can have detrimental effects on the psychological and emotional well-being of individuals. An ethnic minority HCW said in this regard:

*There’s a lot of racism, but it’s covert racism, so you can’t even point it out, you know, you just know that it’s happening because of how differently some people are being treated, how the White staff are being treated from the non-White staff and it’s not just that, it’s just even with for example* [the manager] *be nice to the males, you know, even if you’re an ethnic, but as long as you’re a male it’s fine, it’s more towards the Muslims, you know, at one point there was* [X] *of us and all of us are being treated like rubbish really.* (P15, Female, Other-Arab, AHP, Int)

Often organisational policies, combined with one’s job role and place in the professional hierarchy also created conditions where certain HCWs were discriminated against or were treated unfairly. Emphasising his low pay band, a nurse assistant shared his experiences with accessing PPE and, allocation of duties in the ward:

*It was just we’d been told the PPE in stock is only for physios and the doctors and all that. None of the HCAs, none of the nurses were allowed to wear any FF3 mask or all that and we didn’t have full gowns, you know, we only had like the normal pinnies and gloves and all that. It was really hard, yeah…The thing is, you know, when you are working in a ward and if your higher authorities say that, you can’t question sometimes…I’m a Band 4 and if there’s only two nurses, they always put me on a Covid bay which I have to take in charge of the patient and all other nurses had to take green patients and I’ve always been in there with the red patients. Sometimes I think it’s not fair but I don’t want to blame anybody* (P16, Male, Asian, Nurse, Int).

An ethnic minority participant who had a disability, compared her situation with another White colleague who also had a disability, and the differential treatment she receives to her disability because of her ethnicity:

*People will look at me now, although I tell them that, yes, I’ve been diagnosed with* [learning disability and behavioural condition]…*There’s nothing wrong with you, they say. In the workplace…there’s a young lady, Caucasian lady…and she shouts from the rooftop, I am dyslexic, so what do you expect me to do? Now, I look upon myself, and I thought, if I ever said something like that, what would be said? What doors would open for me if I said that?…But we’ve accepted her, with all her flaws, and it’s fine. But I don’t think that would have been the case for me.* (P17, Female, Black, AHP, Int)

Many participants also shared how they have experienced these acts of microaggression when it comes to their career progression. Several ethnic minority HCWs, particularly women, said how they had been, in their careers, discouraged from applying for higher grade positions or overlooked for promotion even when they were deserving.

*I passed my exams, I got through interviews, I was supported as a junior doctor, you know, I got the job that I wanted, but once in the job and you’re then trying to climb the career ladder, you have got to work twice as hard to prove that you’re as good as a White middle-aged man is. So, to then be shown – then to demonstrate that you’re not just as good as a White middle-aged man is, but that you arebetterthan them, so you’ve got to put in three/four times the amount of work to be able to compete with that man to get the job that both of you applied for.* (P12, Female, Asian, Doctor, Int)

#### Stereotyping

Some ethnic minority HCWs reported the use of negative stereotypes, and feeling that they were being judged and treated in a prejudicial manner because of their social identity. An ethnic minority HCW, who was trained abroad, shared how she experienced discrimination from patients who make assumptions on her capabilities and that she is not adequately qualified based on her skin colour and origin:

*I think it stems more from stereotyping. That’s what I kind of get, that the person maybe has grown up with a perspective of how a black person should be and whether they are really, in spite of the fact you are treating them and you have been qualified and been checked to work as a nurse, still that doesn’t come across in spite of that. That doesn’t come across that you couldn’t have faked yourself or your documentation to reach where you are, so still we feel, I feel that stems from maybe the stereotyping expecting that you don’t know what you’re doing or how, whether you’re that educated to be able to be doing such kind of job.* (P18, Female, Black, Nurse/Midwife, Int)

Some participants reported that countering or questioning patients and colleagues about microaggressions often gets them labelled as ‘trouble-makers’ and perpetuates stereotypes associated with people of colour around aggressive and angry personalities [30]. A female, Black midwife said:

*So, you constantly feel like you’ve got to defend it, and when somebody says something, you can’t just stand back and be like, ‘I’ll just let it ride’. So, I did find myself constantly being like, ‘Well, that’s not right, and do you know why this happens and stuff’. So, you’re constantly explaining everything, but then, that has the effect of then me being labelled the angry black woman and I’ve got a chip on my shoulder*. (P11, Female, Black, Nurse/Midwife, Int)

While ethnicity has been an important factor in HCWs experiencing discrimination, there are other factors at play (such as gender, foreign-accent, or attire) which led to selective targeting of HCWs, by patients and colleagues alike. As one British-born ethnic minority HCW reported:

*I am British, I am of an ethnic minority but I don’t suffer anywhere near as much as my colleagues who are of ethnic minorities who are not – who either don’t have British citizenship or just simply have an accent for a start, are viewed differently by patients and colleagues and, yeah, I suppose are trained elsewhere.* (P19, Female, Other-Arab, Doctor, Int)

## Discussion

We undertook one of the largest qualitative studies in the UK exploring the experiences of HCWs from different ethnicities and roles in the UK, during the COVID-19 pandemic. Our data identifies a multifaceted and complex set of issues influencing HCWs’ experiences and, hence we utilise a methodological framework informed by intersectionality for analysing our results. Our analysis recognises that multiple factors including ethnicity, gender, migration status and hierarchy of roles have led to conditions where HCWs felt they were particularly disempowered, disadvantaged or discriminated against. These perceptions were exacerbated by the intrasectional experiences they faced within the ecosystem they worked in during the pandemic. Whilst in some cases there were deliberate efforts to disempower, disadvantage or discriminate, our research also acknowledges these experiences can occur without those in positions of privilege ever knowing that the other group is at a disadvantage [31]. Our research explores the specific circumstances in these groups and our findings identify disempowerment, disadvantage, and discrimination at various levels of the ecosystem. This intrasectional analysis adds to the previous approaches of intersectionality by demonstrating how individuals’ experiences are linked to wider issues of power and dominance that straddle across and are compounded at multiple levels including unfair system-wide policy responses [32].

Previous research has shown how HCWs’ ethnicity has impacted on their agency, and the disproportionate redeployment of ethnic minorities to COVID ‘hot wards’ [33]. There is also evidence from before the pandemic of the racism and discrimination against HCWs working in the NHS [34–36]. We have also long known that female employees face the ‘glass ceiling’ at work with limited career progression opportunities to top-levels [37, 38]. Our research adds to this evidence-base by showing that ethnicity or gender alone might not disadvantage HCWs from exercising their agency or progressing in their careers, rather it is the intersection of gender, ethnicity, age, migration status and maybe more which is disempowering.

Pressure to deliver care during the pandemic, in environments in which HCWs did not feel safe, has been reported in previous research [39–41]. Our findings, however, demonstrate that the burden of healthcare delivery was disproportionately borne by HCWs in certain social positions. This was in the context of a national strategy (at macro level) to keep the NHS always operating at a certain capacity at all costs [42], which put further pressure on the workforce to continue with an “obligation to treat”. Our research highlights how this obligation to treat (at macro level), coupled with the pressures of employment precariousness, particularly for migrant workers who feared that they had to do what they were told or lose their jobs and/or visa status (at meso/micro level), created conditions where HCWs felt they had less agency. This, in addition to socioeconomic stressors, and socio-cultural differences that HCWs reported prevented them from questioning authority, exemplifies how multiple factors may disadvantage individuals and groups.

Precarious financial status also disadvantaged those HCWs situated within the context of intersecting structures around gender roles, pay and leave, and social security measures. Many of these HCWs, such as single parents and first-generation immigrants, also faced the added hardship of not having access to protective factors such as social support [43, 44]. These inequities in social capital among certain groups need to be taken into consideration while formulating policies at work.

Our research also adds to the literature on microagression and stereotyping based on people’s identities [29, 45]. Data shows that discrimination on the grounds of religion, ethnicity or gender is prevalent in the UK healthcare workforce [46–48], but our study finds that those HCWs who stand at the intersection of all these identities face greater discrimination, at times on a daily basis. HCWs facing such discrimination are at risk of experiencing further disparities in their health or careers [38, 49]. Our qualitative intrasectional analysis provides findings that show the sense of alienation and injustice that discrimination encompasses for some HCWs.

This paper follows on from our original call for more intersectional analysis which can be enabled by specific actions such as: a review of how relevant data are collected and made accessible; a more unified approach to data to enable intersectional analyses; and the integration of a more intersectional approach in equality, diversity, and inclusion policies by employing organisations [50]. To add further, our findings, informed by an intrasectional approach, can offer significant insights in tailoring context-specific policies for the well-being and development of HCWs in the aftermath of the pandemic at all levels of policy making. The pandemic had taken a toll on healthcare systems all over the world and at present many countries, including the UK, are faced with problems of staff burn-out, attrition, strikes and rising sickness absence [51–53]. Our intersectionality-based analysis provides evidence that although macro-level problems, like the pandemic, can affect everyone, the impact it has on certain groups with intersectional marginalised social identities, can be varied and more profound than others. This calls for a shift in policy-making from a one-size-fits-all approach to more nuanced thinking. For instance, policy-makers looking to improve staff retention in the NHS should consider how migrant HCWs from ethnic minority backgrounds would be impacted by changes in national immigration policies which might be missed if all ethnic minority HCWs (including those with British citizenship) were regarded as a homogenous category.

### Limitations

This paper used the data from the qualitative sub-study of UK-REACH. This qualitative research was specifically designed to explore the experiences of a diverse cohort of HCWs’: their perceptions of risks; safety and protection; fears and concerns; and support and coping mechanisms while working during the pandemic. As such, the study was not influenced by an intersectional conceptual framework in its original design. However, considering the diversity of the sample and the context of the study, we as researchers felt that utilising an intersectional and related intrasectional approach to analysing the data will yield novel findings.

As a result, our study provides insight into the lived experiences of HCWs from across the workforce using qualitative methods. While this has yielded rich and nuanced results, statistical associations and generalisations cannot be drawn from the qualitative data. Due to social distancing measures in place at the time, recruitment and data collection had to be conducted remotely using online technology, which might have affected participation and the depth of experiential insights gained from certain groups who may be less proficient in use of or have less access to digital technology.

## Conclusions

Our research demonstrates that multiple intersecting factors including occupation, ethnicity, gender, and migration status had an intrasectional, discriminatory, disadvantageous and disempowering impact on HCWs during the COVID-19 pandemic. Much of the current evidence gathered on inequalities for HCWs during this time lacks an inter- or intra-sectional approach thereby missing the nuanced and different ways in which communities of HCWs are marginalised. Our research offers empirical evidence of how these instances of marginalisation and discrimination are experienced and perpetuated within the healthcare workforce thus paving the way for more holistic approaches to learn and remedy these problems.

## Data Availability

The data for this study consists of interview transcripts of participants that contain potentially identifying and sensitive information. The data cannot be shared publicly due to concerns of participant confidentiality and ethics requirements. Participants consented to the study with the understanding that only de-identified quotations would be made public, not the entirety of the transcripts. Therefore, only illustrative quotes from the transcripts have been included in this paper. Data for this study could be made available upon reasonable request to the UK-REACH Data Access Committee (uk-reach@le.ac.uk), which is the institutional email of the UK-REACH project.
